# Molecular Minimal Residual Disease Detection in Acute Myeloid Leukemia

**DOI:** 10.3390/cancers13215431

**Published:** 2021-10-29

**Authors:** Christian M. Vonk, Adil S. A. Al Hinai, Diana Hanekamp, Peter J. M. Valk

**Affiliations:** 1 Department of Hematology, Erasmus MC Cancer Institute, University Medical Center Rotterdam, 3015 CN Rotterdam, The Netherlands; c.m.vonk@erasmusmc.nl (C.M.V.); adilsalim.alhinai@moh.gov.om (A.S.A.A.H.); d.hanekamp@erasmusmc.nl (D.H.); 2 National Genetic Center, Ministry of Health, Muscat 111, Oman; 3 Department of Hematology, Cancer Center VU University Medical Center, Amsterdam University Medical Centers, 1081 HV Amsterdam, The Netherlands

**Keywords:** acute myeloid leukemia, minimal/measurable residual disease, MRD, next generation sequencing, clonal hematopoiesis

## Abstract

**Simple Summary:**

Although the majority of patients with acute myeloid leukemia (AML) reach a morphologic complete remission after high-dose chemotherapy, the majority of them face a relapse within a few years. Detection of residual cells, persisting in a considerably small amount, has shown to be predictive of impending relapse in multiple studies. Whereas the gold standard in minimal residual disease (MRD) detection in AML is currently based on immunophenotypic approaches, the use of molecular MRD testing to predict AML relapse has been explored extensively in recent years. This review aims to provide an overview of the different studies that improve molecular MRD detection in AML, and to describe the limitations and challenges it faces.

**Abstract:**

Initial induction chemotherapy to eradicate the bulk of acute myeloid leukemia (AML) cells results in complete remission (CR) in the majority of patients. However, leukemic cells persisting in the bone marrow below the morphologic threshold remain unaffected and have the potential to proliferate and re-emerge as AML relapse. Detection of minimal/measurable residual disease (MRD) is a promising prognostic marker for AML relapse as it can assess an individual patients’ risk profile and evaluate their response to treatment. With the emergence of molecular techniques, such as next generation sequencing (NGS), a more sensitive assessment of molecular MRD markers is available. In recent years, the detection of MRD by molecular assays and its association with AML relapse and survival has been explored and verified in multiple studies. Although most studies show that the presence of MRD leads to a worse clinical outcome, molecular-based methods face several challenges including limited sensitivity/specificity, and a difficult distinction between mutations that are representative of AML rather than clonal hematopoiesis. This review describes the studies that have been performed using molecular-based assays for MRD detection in the context of other MRD detection approaches in AML, and discusses limitations, challenges and opportunities.

## 1. Introduction

Acute Myeloid Leukemia (AML) is a stem cell disorder within the hematopoietic system arising from aberrant proliferation of undifferentiated myeloid progenitor cells and is characterized by a considerable clonal and genetic heterogeneity [1]. In recent years, advancements have been made in understanding the genetic and molecular landscape underlying AML [2]. With the emergence of novel and more sensitive techniques, such as whole-genome sequencing and whole-exome sequencing (WGS, and WES, respectively), detailed analyses of the disease have become feasible and more efficient.

At the time of AML diagnosis, a classification and risk assessment is made depending on morphologic, immunophenotypic, cytogenetic and molecular aberrations present in the patients’ bone marrow (BM). The (cyto)genetic markers allow for a classification into different molecular subgroups with distinct prognostic outcomes; favorable, intermediate, or adverse as summarized in the 2017 European LeukemiaNet (ELN) recommendations for diagnosis and management of AML [3]. This classification forms the basis upon which treatment decisions are made, where patients in the adverse-risk group are considered for allogeneic stem cell transplantation and the favorable risk-group patients are not. Treatment is generally started with intensive induction chemotherapy to eradicate the bulk of leukemic cells, after which a majority of the AML patients reach a morphologic complete remission (CR) [4]. However, relapse rates are still high, affecting more than 50% of patients within a few years after diagnosis [5].

Currently, post-treatment analysis is generally conducted morphologically, where CR is defined as <5% blast cells remaining in the bone marrow [4]. However, leukemic cells that reside below this morphologic threshold are unaffected by chemotherapy and have the potential to re-emerge as disease relapse. The ability to detect these small persisting cell populations early on has the potential to guide physicians in deciding to change treatment and prevent patients from relapse [6,7]. Molecular minimal/measurable residual disease (MRD) detection permits a highly sensitive evaluation of an individual patient’s relapse risk and response to treatment, making it a promising prognostic marker in AML.

## 2. Minimal/Measurable Residual Disease

MRD is defined as the persistence of a small number of malignant cells after initial treatment, undetectable by conventional screening methods, yet measurable by more sophisticated technologies. The residual cells are often present without clinical signs or symptoms of the disease, but can potentially be used as a predictive or prognostic biomarker when detected [7]. Several assays are currently available for the detection of MRD, which can be grouped into two different approaches: immunophenotypic, with multiparameter flow cytometry (MFC), and molecular, with real-time quantitative polymerase chain reaction (RQ-PCR), digital droplet PCR (ddPCR) and/or next generation sequencing (NGS). Each of these methods differs in their applicability, specificity and sensitivity of detecting MRD.

### 2.1. Multiparameter Flow Cytometry Approaches

The introduction of MFC in routine diagnostics allowed a more detailed and sensitive examination of BM for both diagnosis and MRD detection of AML [8,9]. MFC-based MRD detection relies on the presence of immunophenotypic aberrant antigen expression where leukemic cells can be discriminated from normal cells by leukemia-associated immunophenotypes (LAIPs). LAIPs can be identified on blast cells and are a combination of a myeloid marker, a normal progenitor antigen and (multiple) aberrantly expressed cell surface marker(s) [10]. 

Two main approaches are used for the detection of MRD by MFC: the LAIP approach, where LAIPs are defined at diagnosis and their presence subsequently monitored at follow-up [11]; and the different-from-normal (DfN) approach, which screens follow-up material on the presence of aberrant LAIPs, irrespective of the LAIP at diagnosis [12]. MFC-MRD is applicable and widely accessible in the majority of AML patients, and in the past decade many laboratories have gained experience in MFC analysis, making it the current gold standard to determine MRD in AML. Its sensitivity is reported to range from 10^−3^ to 10^−5^ [13]. However, accurate assessment is dependent on various factors, including the expertise of trained personnel, making it a highly subjective technique. Hence, standardized MFC data analyses are hard to implement and alternative MRD detection techniques are being explored.

### 2.2. Molecular Approaches

The Reverse-Transcription Polymerase Chain Reaction (RT-PCR), as well as RQ-PCR, can be used for the assessment of MRD in specific subsets of AML, in particular those that harbor fusion transcripts or specific somatic mutations [14]. RQ-PCR is able to accurately quantify the abundance of these genetic aberrations by combining conventional PCR with a fluorophore measuring the amplification of the PCR-product in real time. RQ-PCR strategies generally reach relatively high sensitivities of detection, i.e., 10^−5^–10^−6^ [15]; on average tenfold higher compared to MFC.

The applicability of RT/RQ-PCR is limited to a selected number of recurrent genetic changes in AML. For instance, RQ-PCR is employed for detection of the fusion genes *RUNX1*/*RUNX1T1* and *CBFB*/*MYH11* in core-binding factor (CBF) leukemia’s characterized by a t(8;21) chromosomal translocation or inversion of chromosome 16, respectively [16]. Several studies have reported that molecular MRD of these fusion genes in CR after initial chemotherapy leads to an increased risk of relapse. For example, a prospective study of 278 patients concluded that MRD monitoring by quantitative RT-PCR of the CBF fusion genes can identify patients with an increased risk of relapse [17]. Similar results were found in a prospective study of 198 patients enrolled in the French-CBF 2006 trial [18], as well as in several smaller CBF-AML study cohorts [19,20]. Of note, screening for persistence in CR has not been broadly incorporated in clinical routine due to the incidence of clonal hematopoiesis (CH), a non-malignant expansion of hematologic cells harboring specific recurrent mutations. Hence, AML patients can have low-level fusion transcripts in CR but never relapse, suggesting a state of CH rather than residual leukemia. However, reappearing or rising levels of fusion transcripts have been shown to associate with disease relapse. Identifying mutations associated with CH could aid in predicting subsets of AML patients less likely to relapse.

Along with the CBF-leukemia transcripts, RQ-PCR has been successfully applied to detect MRD in adult AMLs with mutations in the Nucleophosmin (*NPM1*) gene. *NPM1* mutations are among the most frequently observed molecular lesions in AML, occurring in approximately 30% of all patients and in 50–60% of AMLs with normal karyotypes [21]. At present, more than 55 different *NPM1* mutations, generally 4 base-pair insertions, have been observed, of which three types (A, B, and D) account for circa 95% of all cases [22]. The 4 base pair-insertion mutations in *NPM1* are generally stable throughout the course of disease including at time of relapse [23]. However, this may not be the case for all *NPM1* mutant AML patients, since no mutant *NPM1* was detectable in 9% of patients at time of relapse [24]. Succeeding the first study describing the quantitative MRD assessment of *NPM1* mutant AML by RT-PCR [25], multiple additional studies have monitored *NPM1* mutant MRD. More recently, RQ-PCR for *NPM1* mutations in a large cohort of 346 patients demonstrated a clear association of persisting *NPM1* mutations with a greater risk of relapse [26]. These results were in concordance with previous findings where *NPM1* mutations persisting in CR were a strong prognostic marker for the development of disease relapse [24,27,28,29,30,31]. Of note, low levels of *NPM1* mutant MRD are associated with a higher risk of relapse only in the presence of a co-occurring *FLT3* internal tandem duplication (ITD) [32].

In contrast, MRD assessment of DNA MethylTransferase 3A (*DNMT3A*) mutations by RQ-PCR was not predictive of relapse in AML patients. In a cohort of 181 patients that harbored one of two known hotspot mutations in *DNMT3A*; R882H or R882C, transcript levels at multiple time-points were determined. In the majority of patients, the presence of mutant *DNMT3A* in CR did not result in AML relapse, indicating that mutations in *DNMT3A* occur early on in leukemogenesis and that additional mutations in driver genes are required for the development of AML. Thus, hotspot mutations in *DNMT3A* appeared not to be a suitable target for MRD testing in AML [33].

In addition, the overexpression of certain genes can be measured by RQ-PCR and were shown to have prognostic value as MRD marker in AML. Overexpression of the Wilms Tumor 1 (*WT1*) gene, encoding a transcription factor often overexpressed in AML, is most studied in this context [34]. Several studies have applied RQ-PCR for sequential monitoring of *WT1*, and reported an increased risk of relapse associated with elevated *WT1* levels [35,36]. Although molecular assays based on gene transcript levels are applicable for patients without AML-specific molecular markers, they have some limitations. For example, the sensitivity is limited by the expression of the wild type gene in the tissue of interest, leading to an estimated subset of only 13–46% of AML patients with *WT1* expression high enough to serve as MRD marker [36]. In efforts to overcome this, combining quantification of *WT1* with MFC led to an improved prediction of relapse [37].

Molecular MRD in adult AML may also be detected by means of digital droplet PCR (ddPCR); a digital PCR-based assay using absolute quantification of amplified target genes without the need of standard curves. The feasibility of ddPCR in detecting MRD has been tested in several studies and is eligible in particular for *NPM1* mutant AML patients, [38,39,40,41]. In addition, some studies have explored the use of ddPCR for MRD detection of other leukemia-associated mutations, including in *IDH1*/*2* [42,43] or in a subset of different mutations associated with AML [44,45]. Although these studies concluded that ddPCR is a feasible method for predicting relapse using MRD detection in AML with a relatively high limit of detection, larger cohort sizes are needed to confirm these mutations as reliable MRD markers. However, a major limitation of ddPCR is that each assay needs to be specifically designed for every acquired aberration, meaning that in contrast to recurrent mutations in AML, ddPCR would be a less efficient and more laborious approach for rare patient-specific mutations without a standardized assay.

### 2.3. Next Generation Sequencing (NGS) for MRD Detection in AML

Despite the high sensitivity of RT/RQ/dd-PCR-based assays in detecting MRD of AML carrying specific gene fusions or hotspot mutations in driver genes, their applicability is limited to only specific AML subsets due to the unavailability of robust molecular markers in the remaining AML cases. NGS provides a solution by allowing the detection of various and patient-specific gene mutations in a single assay [46]. NGS approaches make use of high-throughput sequencing techniques and refer mainly to several different modern massively parallel sequencing technologies such as WGS, WES and targeted sequencing. These approaches provide DNA sequencing data of whole genomes, whole exomes, or multiple genes, respectively, in a more efficient and less time consuming manner compared to for example Sanger sequencing [47]. Molecular MRD detection using NGS permits a comprehensive and relatively sensitive evaluation of an individuals’ response to treatment, thereby providing potentially important prognostic and predictive information in AML patients. Multiple studies have been performed where detecting molecular MRD in adult AML using NGS is examined (Table 1).

Several early studies have explored the ability of applying NGS for the detection of molecular MRD, initially focusing on selected molecular markers. In 2012, MRD detection based on *NPM1* mutations and *FLT3*-ITD mutations in 20 AML patients demonstrated that NGS can reliably assess molecular MRD status, and showed a 95% concordance with RQ-PCR for mutated *NPM1* [48]. In another study, the potential of *RUNX1* mutations as MRD marker was investigated using deep amplicon sequencing in a prospective cohort of 814 AML patients, with 103 patients eligible for *RUNX1* paired diagnosis-remission analysis. Median residual *RUNX1* mutational burden, defined as 3.61% of variants reads in follow-up, was used to assign patients to two different groups, with one group (<3.61% mutational burden) having a significantly better outcome in terms of EFS and OS [49]. In recent years, several studies have shown that MRD detection by targeting multiple molecular markers using NGS is feasible and associates with response to therapy in AML. In 2015, an NGS-based MRD study was performed on 50 AML patients receiving standard induction chemotherapy [50]. WGS or WES was carried out on AML samples obtained at diagnosis, followed by enhanced deep exon sequencing targeting 264 recurrently mutated genes in paired AML diagnosis and CR samples. Of these patients, 48% had persistent mutations in CR with a variant allele frequency (VAF) of at least 2.5%, and a significantly reduced event-free survival (EFS) and overall survival (OS). This study demonstrated that NGS-based approaches could improve risk stratification of AML patients. Besides AML patients receiving standard chemotherapy, NGS-based MRD detection has also been explored in patients who underwent hematopoietic stem cell transplantation (HSCT). Getta et al. investigated if NGS could be used for MRD detection, in this study defined as mutations present above a VAF of 5% before HSCT. Mutations detected by a panel of 28 genes at diagnosis and prior to allogeneic HSCT were compared with MRD detected by MFC [51]. A concordance of 71% between the two MRD detection assay results was demonstrated, and detectable MRD appeared to be significantly associated with an increased risk of relapse post-transplantation. Patients with MRD detectable with both assays showed the highest risk of relapse, indicating that a multi-gene NGS gene panel can provide additional clinical information compared to MFC alone [51].

In a subsequent study, targeted NGS was performed on bone marrow or peripheral blood samples of 482 AML patients obtained at diagnosis and at CR after induction chemotherapy [52]. By using a gene panel covering 54 recurrently mutated AML genes, there was at least one detectable mutation found in 89.2% of patients at diagnosis. Using the same assay for samples obtained after therapy, 51.4% of patients harbored a persistent mutation with varying rates across genes, and VAFs ranging between 0.02 and 47%. The detection of a persistent mutation in CR was significantly associated with a higher incidence of relapse. Interestingly, persisting mutations in genes associated with age-related clonal hematopoiesis (CHIP); *DNMT3A*, *TET2*, *ASXL1* (*DTA*), were among the most common, and were frequently present at a relatively high VAF. Patients with only *DTA* mutations persisting in CR were significantly less likely to develop a relapse, whereas patients that harbored a persisting mutation in other genes than *DTA* were associated with an increased risk of relapse, a reduced relapse free survival (RFS), and a reduced OS, also in multivariable analyses [52].

Around the same time, Morita et al. [53] investigated whether MRD status in CR could predict an impending relapse in a cohort of 131 AML patients. A gene panel consisting of 295 genes was used to evaluate mutations in pre-treatment samples, revealing at least one mutation in 93% of patients. BM samples of patients that reached CR at 30 days post induction chemotherapy were sequenced. Different VAF cut-offs (2.5%, 1.0%, and undetectable) were used to examine the association between clinical outcome and clearance of mutation after therapy. Persistent mutations with VAF <1% were associated with a substantial better OS compared to patients with higher VAFs. Patients with no detectable mutations post-therapy showed significantly better EFS [53]. These prognostic associations were stronger when cases were excluded with persisting mutations in *DTA* genes only.

In another study of 104 AML patients receiving allogeneic HSCT, MRD was assessed pre- and post-HSCT [54]. A panel targeting 84 genes was used on samples obtained at diagnosis, pre- and post-HSCT. At diagnosis, 86.5% of patients harbored at least one mutation. Mutation clearance was found in 44.5% of patients pre-HSCT, with a further reduction after transplantation. Although patients with a VAF of 2% at pre-HSCT had a worse OS, no association was found with relapse incidence. Bone marrow samples were collected 21 days after transplantation and sequenced utilizing a computational error correction approach, with a cut-off of 0.2% VAF. Detection of MRD post-HSCT was significantly associated with an increased risk of relapse and a decreased OS compared to AML patients with undetectable MRD [54].

The use of a high-sensitivity targeted NGS-based MRD detection assay was again investigated by using a gene panel covering 46 genes on 116 pre-HSCT AML patient samples [55]. In this analysis at least one potential MRD marker was found in 93% of AML cases. Of these patients, 45% were found to have detectable persisting mutations with a median VAF of 0.33%. In order to increase the sensitivity, error-corrected sequencing (ECS) with unique molecular indices (UMIs) was applied, enabling detection with a sensitivity of <0.02%. Residual molecular MRD measured at CR was found to be an independent predictor of relapse and survival by multivariate analysis [55].

In a similar, retrospective study, 42 AML patients were sequenced using a 42 gene panel at diagnosis, and before allogeneic HSCT time points. With a relatively high limit of detection of 0.5%, persistent mutations in pre-transplant samples were found to be a significant predictor of leukemic relapse and survival [56].

In 2019, Balagopal et al. explored a hybrid-capture error-corrected NGS method with the incorporation of UMIs on post-HSCT samples that were previously evaluated as negative by engraftment studies. By utilizing the UMIs, mutations at a VAF of <0.1% could be reliably detected in 22 frequently mutated genes in AML. With this improved sensitivity, previously undetected residual mutations associated with an eventual relapse were found in 18 out of 30 AML patients [57].

Hourigan and colleagues [58] examined blood samples from a cohort of 190 pre-transplant AML patients who reached morphologic CR and received allogeneic HSCT. In this study, the clinical impact of myeloblative conditioning (MAC) or reduced intensity conditioning (RIC) regimens for AML patients with molecular MRD in preconditioning blood before transplantation was investigated. Ultra-deep ECS was performed for 13 commonly mutated genes in AML, and patients were randomly allocated to either MAC or RIC. DTA mutations were among the most commonly detected in this study but had limited prognostic value. For AML patients with a detectable non-DTA mutation pre-transplant, they observed significant differences in relapse rates (19% vs. 67%; *p* < 0.001) and OS (61% vs. 43%; *p* = 0.02) between patients with MAC or RIC, respectively. This study provides evidence that MAC may result in highly improved outcome for AML patients with pre-transplant molecular MRD [58].

More recently, Heuser et al. [59] assessed whether MRD monitoring of non-DTA mutations would be of prognostic value regarding relapse and overall survival in post-allogeneic HSCT AML patients. In a cohort of 154 AML patients, 138 had a mutation present at diagnosis (90%). Using an error-corrected based NGS assay, residual disease was detected in 25%. In AML patients harboring residual DTA mutations no effect was observed on relapse- and survival rates. In contrast, the presence of MRD defined by non-DTA mutations was found to be an adverse predictor for both relapse and survival, indicating that MRD defined by non-DTA mutations is of prognostic value for post-allogeneic HSCT patients [59].

In another recent study [60], a targeted NGS approach in 335 AML patients was used to assess MRD at two different time points: in CR and after consolidation therapy. A total of 54 genes associated with AML was studied with the exception of mutations in DTA, CEBPA and FLT3-ITD, due to either their association with CH (DTA) or limited sequencing sensitivity (CEBPA and FLT3-ITD). Detectable MRD was defined as variants with a VAF higher than 2 standard deviations from the mean background error, and was detectable in 46.4% of AML patients in CR and 28.9% after consolidation. MRD at both time points was associated with an increased incidence of relapse, as well as decreased OS, also in multivariate analysis. The prognostic impact of detectable MRD after first consolidation therapy was higher compared to that in CR. AML patients without persisting mutations only after consolidation had similar outcomes as patients without MRD before and after consolidation. [60].

Recent assessment of molecular MRD in a study including 132 AML patients undergoing allogeneic-HSCT revealed prognostic value of persistent mutations at both pre- and post-HSCT. The presence of any persistent mutation was associated with a higher risk of relapse and decreased OS. In contrast to previous findings, persistence of isolated DTA mutations in CR was also associated with post-transplant relapse [61].

The suitability of DTA mutations as MRD marker in AML was further evaluated in a recent study including 68 AML patients harboring at least one mutation in DTA genes at diagnosis. No association was found between persisting DTA mutations in CR before HSCT and relapse or OS. Interestingly, when hotspot mutations in DNMT3A (R882) and ASXL1 (G646fs*12) were excluded, the remaining AML patients appeared to have a worse clinical outcome. As opposed to previous findings, these results may indicate that specific non-canonical mutations in *DTA* genes could be suitable MRD markers in AML [62]. Larger AML cohorts will be needed to confirm these findings.

The impact of CH-associated mutations in AML patients harboring an NPM1 mutation has recently been studied in a retrospective cohort of 150 AML patients [63]. In addition to aberrations in DTA genes, mutations in SRSF2, IDH1 and IDH2 were defined as mutations associated with CH. Persistence of these mutations in CR was shown not to be associated with worse EFS and OS, which indicates that these mutations represent a pre-malignant state where the acquisition of additional mutations is needed for the development of AML, similar to what has been proposed for *DTA* mutations [52], and that the acquisition of *NPM1* mutations is a later event in the formation of leukemia [63].

### 2.4. Combining NGS and MCF for MRD Detection

Currently, the gold standard in MRD testing is MFC. While both immunophenotypic and molecular techniques have their own principles, and therefore their own limitations, limited studies are published where multiple methods were applied and compared [51,64,65]. Studies comparing NGS and MFC in 62 and 340 patients showed that the two techniques had an overall concordance of ~70% [51,52]. Moreover, patients with detectable MRD by both assays had the highest risk of relapse. A discordance was seen in a fraction of 64/340 (19%) of AML patients with detectable MRD by NGS only, and for 41/340 (12%) of patients with detectable MRD by MFC only. Interestingly, AML patients with discordant results between NGS and MFC had worse outcomes compared to patients without detectable MRD by both techniques [52].

More recently, Patkar et al. [66] evaluated MRD in 201 AML patients by both techniques after induction- and consolidation therapy. For NGS, the limit of detection was a VAF of 0.05%, and detection of MRD was significantly associated with inferior outcome for both time points. Detection of MRD by NGS was equivalent to MFC in >80% of patients, with discrepancies in only a fraction of AML patients, where prediction of outcome with MRD by NGS seemed to be superior to those with MRD by MFC [66].

## 3. Challenges Related to MRD Detection by NGS

### 3.1. Sensitivity & Specificity

The most challenging limiting factors in AML MRD detection by NGS are the sensitivity and specificity of the sequencing assays. Due to intrinsic properties of the sequencing devices such as cross-talk between clusters and phasing effects [67], or during PCR amplification at the library preparation stage, errors are introduced that influence the ultimate low-level base calling by NGS. Whereas in usual clinical settings, NGS can be reliably used to detect disease-specific mutations with VAFs of >1%, MRD applications generally desire detection of VAFs in the range of 0.01–0.5%. Most sequencing devices harbor an error rate of approximately 1%, meaning that the limit of detection for MRD applications could clearly pose a problem [68]. Of note, sensitivity issues for MRD detection can be mutation specific. For example, insertions in *NPM1* and *FLT3*-ITDs are easily discriminated from noise and can be detected at low levels. However, low-level detection of persistent *NPM1* mutations may not directly associate with increased risk of AML relapse [24,32], whereas late transforming events, such as mutations in *FLT3* may associate when detected at low level.

In order to overcome possible issues with sequencing error rates, ECS methods have been introduced. This approach incorporates unique molecular identifiers (UMIs) to the DNA targets during library preparation [68]. These UMIs are short sequences (~3–16 nucleotides) that are used to trail back the original molecular input. Incorporation of UMIs allows for the differentiation between sequencing errors and true mutations in the variant reads produced during the sequencing process. The UMIs are incorporated during the early stages of the library preparation to minimize the effect of errors produced in the initial PCR cycles, while also enabling the removal of PCR duplicates. After sequencing, the resulting reads are grouped based on identical UMI sequences into read families. A variant that is present in all reads within a read family indicates a true mutation, whereas if this variant is only present in a subset of the reads, the variation was presumably caused by a sequencing error [46].

The incorporation of UMIs in the genomic DNA as a form of error-correction for the detection of MRD in AML has been put successfully into practice before in a wide variety of studies, such as in the previously mentioned studies by Thol et al. and Balagopal et al. [55,57]. For practical applications different commercial UMI-guided NGS panels are available, such as ArcherDx, smMIPS, New England BioLabs NEBNext Direct, and xGen Dual Index UMI, among others. Although these panels use a similar approach, they can differ in size of UMIs and whether single or double (duplex UMI) DNA strands are tagged with UMIs. While duplex UMI NGS methods result in a reduced error rate, higher sequencing coverage and costs are required, which makes it currently less appealing to implement duplex UMI NGS methods for clinical use [46]. Alternatively, ECS can be achieved by using biochemical or computational approaches. Biochemically, a proof-reading polymerase enables a last verification step to check whether the right nucleotide is incorporated before synthesis [69]. Computationally, sequencing reads with low quality and mapping scores can be removed since they indicate a high error rate, or a background error model can be used to determine statistically if a variant is expected to be caused by a sequencing artifact or not [70].

Next to intrinsic sequencing errors, the event of index hopping can negatively influence the downstream analysis of NGS MRD detection, which can occur as a result of multiplexing samples in a single NGS run. Index hopping refers to the contamination of prepared libraries with similar index sequences, resulting in the miss-assignment of reads to samples or patients. Index hopping rates were found to be between 0.2 and 6%, depending on the type of library preparation [71], which would obviously affect MRD detection. A solution to this particular problem is the use of non-redundant dual indexing during library preparation, allowing for the ability to filter reads with unexpected index combinations [71,72].

### 3.2. Distinguishing Leukemia from Clonal Hematopoiesis

**Figure 1:**
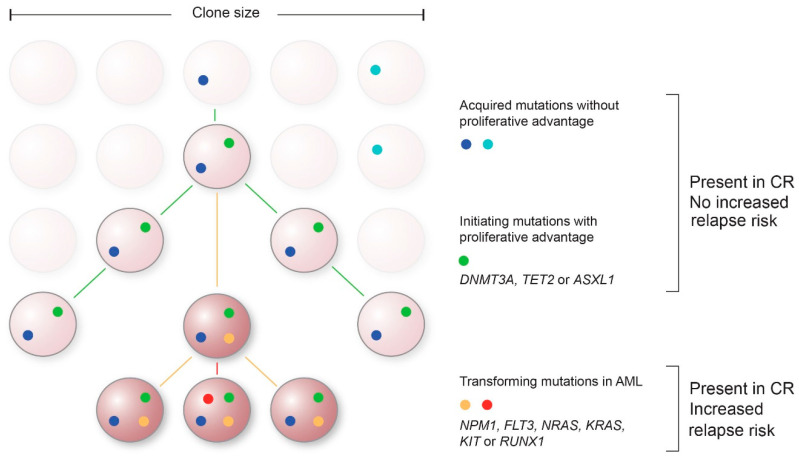
Proposed model of acquisition of mutations over time and their contribution to AML. Mutations without proliferative advantage will occur over time in hematopoietic progenitor cells ((light)blue) that do not contribute to leukemogenesis. Subsequently, initiating clonal hematopoiesis-associated mutations with proliferative advantage can arise, such as mutations in *DNMT3A, TET2* and *ASXL1* (*DTA*) (green). These mutations when persisting in complete remission (CR) typically do not show an association with an increased risk of relapse. Late event mutations in genes such as *NPM1, FLT3, NRAS, KRAS, KIT,* or *RUNX1* are representative for transformation to AML (non-*DTA* mutations, red/yellow). Persistence of these non-*DTA* mutations in CR are associated with an increased risk of relapse.

An obstacle in current molecular MRD approaches is to find the molecular variants that represent and are specific to leukemic cells with the capability to relapse. In addition to mutations driving the leukemia and subsequent relapse, hematopoietic cells can acquire somatic mutations that result in a proliferative advantage in the absence of a hematological malignancy [73] (Figure 1). This clonal expansion of hematopoietic cells arising from one HSC is of particular interest in molecular MRD testing, since the most common mutations associated with CH are also found in leukemia progenitor cells in patients with AML and Myelodysplastic Syndrome (MDS) [74].

Although CH can be a precursor state of leukemia, it is not classified itself as a hematological disorder. The main risk factor for developing CH is aging, with an incidence in the general population of 10–15% in people aged 70 or older and 30% at the age of 85 [73]. Persistence of somatic mutations occurring in the *DNMT3A*, *TET2* and *ASXL1* genes was found to be indicative of a state of CH rather than residual leukemia post-therapy [52,58,59]. Whether persistence of mutations in other genes in age-related CH, such as *JAK2*, *SF3B1*, *SRSF2*, *PPM1D*, *CBL*, *IDH1* and *IDH2*, are associated with an increased relapse risk is currently unknown due to the relative low incidence of these mutations in AML. Enlarging the AML cohorts could bypass this problem. Recently, Cappelli et al. revealed that mutations in *SRSF2*, *IDH1* and *IDH2* in CR represented a state of CH in mutant *NPM1* AML without any association to increased risk of relapse [63]. Another option is to analyze the presence and VAF of mutations at multiple time points, where persistent mutations at constant VAF levels in CR might indicate a state of CH rather than residual leukemia.

In addition, the order in which mutations arise might be of interest in distinguishing leukemia from CH (Figure 1). The first acquired mutations are not necessarily the ones that cause the AML relapse, but may represent a state of CH. Additional mutations are then required to drive the transition from a pre-malignant state to AML. For example, mutations in *DNMT3A* are often acquired early in AML evolution, but are not sufficient to develop leukemia [75]. Additional mutations in genes such as *NPM1*, *FLT3*, or *RAS* are needed for these cells to transform and become oncogenic. Analyses of the VAFs of sequentially acquired mutations provide information of the clonal evolution in AML; mutations with a higher VAF are likely to have been acquired prior to mutations with a lower VAF. Therefore, molecular MRD testing should be aimed at these later mutational events, since they are more likely to represent residual disease. Improved prior recognition of these late event mutations can lead to smaller and more efficient gene panels, thereby enabling the possibility for deeper sequencing with increased sensitivity.

### 3.3. NGS Design Challenges

AMLs harbor on average 13 mutations per exome, while only a subset of these mutations occur in genes recurrently mutated (Cancer Genome Atlas Research Network, 2013). Designing NGS assays that are able to detect all, often patient specific, mutations can therefore be challenging. One method to improve this, is to increase the width of the assay by performing WGS or WES sequencing at AML diagnosis. This enables the detection of a broad range of genomic aberrations including point mutations, insertions/deletions (indels), copy number variations (CNVs) and structural rearrangements of the entire genome or exome in a single run. Although these NGS-based approaches could detect all possible variants, there are currently several limitations to prevent their use for routine MRD testing. Both WGS and WES have a relatively low sensitivity compared to targeted sequencing, and the number of samples to be sequenced in a single run is limited while the data sets are substantial, making them more expensive to use in clinical practice. However, for NGS-based MRD detection, WGS with a relatively low coverage could potentially be utilized to increase the number of targets, facilitating the distinction of residual mutations from noise, thereby potentially increasing the sensitivity of the assay (10^−5^) [76].

An alternative to WGS or WES is targeted sequencing where only a fixed set of genes is being analyzed. This approach reduces sequencing costs and allows sequencing at a higher sensitivity. However, data will be limited to specific genes, and novel gene mutations may be missed. Furthermore, sequencing of two frequently mutated genes in AML; *CEBPA* mutations and *FLT3*-ITDs by using targeted gene panels is challenging for different reasons. *CEBPA* is a GC-rich single exon gene, making it difficult to amplify using PCR and sequence. *FLT3*-ITD mutations, on the other hand, can vary greatly in position and length, with insertions ranging from 3 to 400 basepairs, making it challenging to align the reads to a reference genome. Of note, an open-source analysis program called GetITD does enable high-quality alignment of NGS reads to wild type *FLT3* resulting in an improved identification of insertions [77].

### 3.4. Epigenetics

A major limitation of conventional targeted NGS for low-level disease detection is a maximum level of depth that can be reached to effectively distinguish mutations with low VAFs from background noise [76]. A possibly more efficient method is to look at broader patterns specific to AML, which require a lower level of sensitivity to still enable detection of MRD [76]. In this aspect, an interesting approach is to look at epigenetic changes specific to AML, such as DNA methylation patterns, which are often more robust and widespread across the genome.

Methylation is one of the most studied epigenetic mechanisms, and is involved in multiple biological processes, including regulation of gene expression. An aberrant methylation pattern can result in inhibition or activation of genes and can ultimately contribute to the formation of tumors [78]. In contrast to point mutations, alterations in methylation occur more often in clusters; at CpG islands, i.e., regions rich with CpG dinucleotides, which are often located near the promoter region of a gene. Detection of methylation by NGS requires a distinction between methylated and non-methylated cytosine molecules. By using an enzyme-based or a bisulfite conversion kit, methylated cytosines are transformed into uracils, which will eventually be read as thymines after sequencing. The detection of these aberrant methylation patterns in tumor tissues enables a better distinction of tumor from normal tissue in comparison to NGS MRD-based assays on single base-pair substitutions.

Aberrant DNA methylation patterns have been observed in AML patients with mutations in *IDH1*, *IDH2* and *TET2*, where differentially methylated regions (DMRs) were found in 45 genes [79]. All DMRs were hyper-methylated, indicating a specific epigenetic signature in AML patients harboring these mutations [79]. In an MRD setting, a limited number of studies focusing on methylation patterns have been documented. In 2007, a study found that the presence of different methylation patterns in CR in *p15* and *ERα* was associated with a higher risk of relapse [80]. More recently, four aberrantly methylated CpG sites in AML were studied, but the clinical relevance for MRD detection was not established [81].

### 3.5. Sampling of DNA: Peripheral Blood versus Bone Marrow

The most common and relevant source for MRD detection in AML is the bone marrow. However, since the procedure for extracting material is much more invasive for patients, DNA derived from peripheral blood (PB) should be considered as an attractive alternative, particularly for sequential monitoring. Nowadays, the majority of clinical studies on the impact of MRD in AML are based on BM samples since this provides an increased sensitivity of approximately 1-log in detecting MRD levels compared to PB. Besides, PB is not yet recommended in the ELN2017 guidelines as source for MRD testing [9]. Nevertheless, several studies have explored its use as input for the detection of residual disease.

In 2005 already, the use of PB as input was first tested by *RUNX1*-*RUNX1T1* RQ-PCR in AML patients with a t(8;21) translocation. When comparing BM and PB samples, a similar sensitivity was found, indicating that PB is a suitable source for the detection of MRD in these patients [82]. However, in a large cohort study of CBF-AML, it was shown that the assays on PB DNA did not detect MRD as efficiently as to those on BM with up to 40% of patients showing detectable MRD in BM but undetectable in PB [17]. In addition, Ivey et al. (2016) monitored mutant *NPM1* levels in both BM and PB samples obtained after each cycle of chemotherapy from 346 patients with *NPM1*-mutated AML. They demonstrated that prediction of survival was more effective in PB samples, suggesting that the right source of MRD assessment can be dependent on the type of assay, regimen, and time point [26]. In parallel, PB-MRD assays have been analyzed using MFC. An early study in 50 AML patients using MFC found a significant concordance between BM and PB MRD levels after induction and consolidation therapy, indicating that assessing MRD status with PB can provide prognostic information [83]. Similar results were observed in a larger cohort of 114 AML patients, where paired BM and PB samples were tested for the presence of MRD by MFC. Although the sensitivity was higher in BM samples, PB samples had a higher specificity [84]. More recently, MRD was assessed in BM and PB samples of 209 AML patients. In 83% of patients with detectable MRD in BM samples, the use of PB samples led to detectable MRD as well, indicating a strong concordance between the two. Nonetheless, although PB allows for serial monitoring, BM is currently still the advised input source for MFC-MRD testing due to its higher sensitivity [85].

In addition to PCR- and MFC-based assays, several studies have looked into the use of PB in NGS-based methods. In a retrospective analysis of NGS-based MRD with serial PB and BM samples of 12 AML and 8 MDS patients after HSCT, similar results were obtained with PB and BM suggesting that both could be used for NGS-based MRD in AML. However, the size of this AML cohort was limited, and confirmation using larger sample sizes is needed [86]. Contrarily, in another study, discrepancies in leukemic driver mutations were seen between PB and BM samples due to a shortage of leukemic blasts in the blood. Therefore, they recommended to use BM samples to monitor MRD in AML [87]. As described before, a study by Hourigan et al. used ultra-deep UMI-guided ECS to determine MRD status in frozen blood samples of AML patients in CR. The results indicated that PB can also be used to predict patient outcome by an NGS-based MRD assessment with a more sensitive NGS assay [58]. In addition, a targeted NGS-based study using circulating cell-free DNA (ccfDNA) derived from PB and BM samples was tested for the existence of somatic mutations at diagnosis and in CR of 22 AML patients. Interestingly, some persistent mutations were only detected in ccfDNA, indicating that ccfDNA from PB can give complementary information to BM [88].

Overall, although the use of PB as source for MRD testing seems promising in several studies, its utility remains to be validated within a larger cohort of AML patients, possibly with different MRD thresholds, before it can be considered for routine clinical MRD testing.

### 3.6. Single Cell Approaches

Another challenge in molecular MRD testing is to deconstruct the complex genetic heterogeneity that accompanies AML in the MRD setting. During tumor evolution cells may acquire additional genetic abnormalities, resulting in sub-clonal tumor populations. NGS on the bulk of the tumor cells does not take this clonal architecture into consideration and may miss rare variants occurring in small subsets of cells. Moreover, bulk sequencing is incapable of characterizing changes in clonal diversity over time, making it difficult to interpret information about tumor evolution and its correlation with relapse. As AML may evolve linear or in a complex branched clonal architecture, the application of single-cell sequencing (SCS) can provide a better understanding of the molecular landscape of AML at diagnosis as well as during treatment.

Single-cell analyses can be performed by a commercially available technique, such as the MissionBio Tapestry, 10× Genomics, and Fluidigm among others. However, these techniques are currently met with several limitations, including high allelic dropout rates, small gene panels, a limited single-cell throughput [89], and as SCS is a relatively new approach in AML MRD detection, not many studies have been performed yet.

Recently, some studies have explored the efficiency of using single-cell genotyping to investigate clonal evolution and detect MRD in AML using the MissionBio Tapestry. An increased sensitivity of SCS compared to bulk sequencing with the detection of MRD at 0.12% was shown [90]. In addition, SCS revealed information about the clonal evolution in 14 AML patients, making it easier to distinguish mutations associated with CH [90]. In a larger exploratory study using SCS into clonal evolution of AML, 123 AML patients were sequenced at different time points. It was shown that by using this technique, differences can be observed in combinations of mutations that lead to clonal dominance, and that expansion of minor clones could lead to a change in the clonal architecture [91]. A recent addition to SCS is the incorporation of immunophenotypes, by simultaneously sequencing mutations and identifying cell-surface protein markers of AML clones. Only a few studies have put this technique into practice yet. Miles et al. observed that *CD11b* expression co-occurred with sub-clones harboring a *RAS* mutation [91]. Another recent study explored the utility of proteogenomics in three AML patients, and concluded that it can potentially improve precision medicine in AML [92].

Altogether, SCS is a promising method for the detection of MRD in AML, with the potential to detect MRD cells, deconstruct the clonal architecture, and study the clonal evolution over time.

## 4. Future Perspective of Molecular MRD Detection in AML

Molecular monitoring of MRD in AML patients has recently become more prominent. Since the most widely used molecular technique (i.e., RQ-PCR) has limited applicability for only subsets of AML patients, there is an urgent need to utilize NGS for MRD monitoring. Although still in development, NGS MRD seems to have clear additive values for molecular MRD monitoring. Studies have shown that it can be applied to virtually all AML patients, predict the risk of relapse after therapy, determine patient-specific prognosis, aid in assigning consolidation treatment strategies following completion of standard therapy, and monitor the efficacy of treatment.

In the last decade multiple studies have emerged that explored the use of NGS-based methods to detect MRD in AML. Although all studies underlie the potential clinical utility of NGS-MRD detection, most studies were performed in relatively small cohorts, making it difficult to determine the value of the rare variants as targets for MRD analyses. Prior to being able to fully implement MRD assessment by NGS in routine clinical practice, several additional issues need to be addressed: most importantly improvement of the sensitivity and specificity of molecular assays, and a better distinction between CH and leukemic transforming mutations. Finally, harmonization will be essential to allow accurate comparison of NGS-based MRD results among centers and trials [9]. Consensus should be accomplished in various aspects such as selection of the most relevant molecular markers, sequencing approaches, sampling tissue (BM or PB), and timing of sampling.

## Figures and Tables

**Table 1:** Next Generation Sequencing Studies for MRD Detection in adult AML.

Author	Year	NGS Approach	Cohort Size (*n*)	Mean Coverage	Threshold MRD (VAF)	MRD Mutations	Key Finding
Thol [48]	2012	Amplicon sequencing of *NPM1* and *FLT3*-ITD mutations	20	7758× *NPM1*) 15,278 (*FLT3*)	0.29 for *NPM1* (allelic ratio)	*NPM1* and *FLT3*-ITD	Using NGS, MRD can reliably be assessed for *NPM1* mutations. *FLT3*-ITD clones can be assessed in one single assay
Kohlmann [49]	2014	Amplicon deep sequencing of *RUNX1*	103	844×	1%	*RUNX1*	Detection of residual *RUNX1* can distinguish distinct risk groups based on mutational load
Klco [50]	2015	WES or WGS at diagnosis, followed by deep exon sequencing of 264 recurrently mutated genes in AML	50	543× or 14,780×	5%	13 recurrently mutated genes in AML	Detection of residual mutations associated with AML correlate with increased risk of relapse and decreased OS
Getta [51]	2017	Targeted sequencing using a 28-gene amplicon capture-based panel	104	Unknown	5%	28 recurrently mutated genes in AML	MRD can be detected by NGS using a multi-gene panel before allogeneic HSCT. Detection of MRD by both MFC and NGS was associated with the highest risk of relapse
Jongen- Lavrencic [52]	2018	Targeted sequencing using a 54-gene NGS panel	482	3500×	0.02%	non-*DTA,* persistent mutations found in 27 genes	Detection of persisent mutations in CR associated with CH(*DTA*) did not correlate with an increased risk of relapse
Morita [53]	2018	Targeted capture-based deep sequencing using a 295 gene panel	131	575×	1.0%	35 Recurrently mutated genes in AML/non-*DTA* mutations	Detection of persistent mutations at day 30 after treatment with low VAF (< 1%) was associated with improved EFS and OS. This was enhanced when *DTA* mutations were removed from the analysis
Kim [54]	2018	Targeted sequencing using a 84 gene NGS panel	104	1725.6×	0.02%	Persistent mutations in unknown number of commonly mutated genes in AML	Detection of MRD at 21 days post-HSCT was associated with an increased risk of relapse and decreased OS
Thol [55]	2018	Targeted sequencing using a 46 gene NGS custom amplicon panel with UMI-based ECS	116	6,100×	0.016%	24 Recurrently mutated genes in AML, *NPM1* and *DNMT3A* were excluded	Detection of MRD using NGS with UMI-based ECS before allogeneic HSCT is predictive for relapse and OS
Press [56]	2019	Targeted sequencing of a 42 custom gene panel, including recurrently mutated genes in AML	42	1900×	0.50%	16 recurrently mutated genes in AML	Detection of MRD in pre-HSCT is significant predictor for relapse and OS
Balagopal [57]	2019	Targeted capture-based sequencing using a 22 gene NGS panel with UMI-based ECS	30	10,000×	0.1%	12 recurrently mutated genes in AML	The inclusion of UMIs allows for a highly sensitive MRD detection, effective in predicting a relapse at post-HSCT in AML patients
Hourigan [58]	2020	Targeted ultra-deep sequencing of 13 commonly mutated genes in AML, with UMI-based ECS on PB samples	190	41 × 10^6^	0.001%	13 recurrently mutated genes in AML / non-*DTA*	MRD detection in AML patients pre-HSCT who underwent myeloblative conditioning had lower relapse rates and higher OS compared to patients undergoing reduced intensity conditioning regimens. DTA mutations had no prognostic value
Heuser [59]	2021	Amplicon based ECS for 46 commonly mutated genes in AML	154	526,161×	0.048%	non-*DTA* mutations included in panel	Detection of residual non-*DTA* mutations was highly predictive for relapse and associated with decreased OS in multivariate analysis for post-allogeneic HSCT AML patients
Tsai [60]	2021	Targeted sequencing of 54 genes associated with AML at diagnosis, CR, and after consolidation chemotherapy	335	10,550×	0.3%	42 non-*DTA* mutations included in panel (except for *CEBPA* and *FLT3*-ITD)	Detection of residual non-*DTA* mutations after consolidation therapy has more prognostic value than detection after induction therapy
Kim [61]	2021	Amplicon-based sequencing of 67 genes in a custom NGS panel at three timepoints (diagnosis, pre-HSCT, post-HSCT)	132	2406×	0.01%	All mutations included in panel, *DTA* and CHIP mutations at pre-HSCT	Persistent *DTA* and CHIP-associated mutations at pre-HSCT are associated with increased risk of relapse post-HSCT
Jentzsch [62]	2021	Targeted amplicon sequencing of *DNMT3A* and *ASXL1*	68	Unknown	0.0001%	Canonical mutations in *DTA: DNMT3A* (R882) and *ASXL1* (G646fs*12)	Exclusion of detection of residual hotspot mutations in *DNMT3A* (R882) and *ASXL1* (G646fs*12), leads to a worse clinical outcome in AML patients
Cappelli [63]	2021	Targeted sequencing of 63 genes associated with hematologic malignancy at diagnosis, CR, and relapse, validation using WGS	150	Unknown for panel sequencing, 100× for WGS	1%	non-*DTA* – *SRSF2* -*IDH1*/*2 (CHOP-like mutations)*	In addition to *DTA*, mutations in *SRSF2*, *IDH1*, and *IDH2* were not associated with a worse prognosis in *NPM1* mutated AMLs
Patkar [66]	2021	Capture-based sequencing of 34 genes using a custom NGS panel with the inclusion of UMI-based ECS	201	14,728×	0.05%	*NPM1, FLT3, NRAS, KIT, IDH1/2, WT1, RUNX1, GATA2, U2AF1, PHF6*	Panel-based ECS is highly concordant with MFC techniques for the detection of MRD

Note: Adjusted and supplemented from Yoest et al. [46].
